# Identification of potential target genes of non-small cell lung cancer in response to resveratrol treatment by bioinformatics analysis

**DOI:** 10.18632/aging.203616

**Published:** 2021-10-11

**Authors:** Peng Gao, Guanghui Ren

**Affiliations:** 1 Institute of Microvascular Medicine, Medical Research Center, Shandong Provincial Qianfoshan Hospital, The First Affiliated Hospital of Shandong First Medical University, Jinan, Shandong 250014, China; 2 Shandong Provincial Key Laboratory of Animal Resistant, School of Life Sciences, Shandong Normal University, Jinan, Shandong 250014, China

**Keywords:** non-small cell lung cancer, resveratrol, bioinformatics analysis, ANPEP, ITGAL, CD69, PTPRC

## Abstract

Non-small cell lung cancer (NSCLC) is the most common type in lung cancer in the world, and it severely threatens the life of patients. Resveratrol has been reported to inhibit cancer. However, mechanisms of resveratrol inhibiting NSCLC were unclear. The aim of this study was to identify differentially expressed genes (DEGs) of NSCLC treated with resveratrol and reveal the potential targets of resveratrol in NSCLC. We obtained mRNA expression profiles of two datasets from the National Center for Biotechnology Information Gene Expression Omnibus (NCBI-GEO) and 271 DEGs were selected for further analysis. Data from STRING shown that 177 nodes and 342 edges were in the protein-protein interaction (PPI) network, and 10 hub genes (*ANPEP*, *CD69*, *ITGAL*, *PECAM1*, *PTPRC*, *CD34*, *ITGA1*, *CCL2*, *SOX2*, and *EGFR*) were identified by Cytoscape plus-in cytoHubba. Survival analysis revealed that NSCLC patients showing low expression of *PECAM1*, *ANPEP*, *CD69*, *ITGAL*, and *PTPRC* were associated with worse overall survival (OS) (*P* < 0.05), and high expression of *SOX2* and *EGFR* was associated with worse OS for NSCLC patients (*P* < 0.05). Overall, we identified *ANPEP*, *CD69*, *ITGAL*, and *PTPRC* as potential candidate genes which were main effects of resveratrol on the treatment of NSCLC. *ANPEP*, *ITGAL*, *CD69*, and *PTPRC* are all clusters of differentiation (CD) antigens, might be the targets of resveratrol. The bioinformatic results suggested that the inhibitory effect of resveratrol on lung cancer may be related to the immune signaling pathway. Further studies are needed to validate these findings and to explore their functional mechanisms.

## INTRODUCTION

Lung cancer has the second highest incidence and the highest mortality in the world (https://www.iarc.fr/faq/latest-global-cancer-data-2020-qa/) [1]. Non-small-cell lung cancer (NSCLC) accounts for about 80% of all lung cancer. Chemotherapy is the primary treatment of lung cancer [2] and great progress has been made in chemotherapy treatment, but the prognosis is still poor [1]. Therefore, it is imperative that searching for effective targets for the diagnostic of NSCLC.

Resveratrol is a natural polyphenolic phytoalexin that is extracted from grapes, mulberries, and peanuts [3]. As an activator of sirtuin 1 (SIRT1), resveratrol has been reported to affect many physiological processes including senescence, and many diseases including metabolic syndrome, hypertension, Alzheimer’s disease, cardiovascular diseases, and inflammatory disease [4, 5]. The potential of resveratrol on cancer treatment has been reported and a large number of studies have confirmed that resveratrol has a good inhibitory effect on various tumors including NSCLC [6]. Wang et al. found that resveratrol might inhibit proliferation but induce apoptosis and autophagy via inhibiting Akt/mTOR pathway and activating p38-MAPK pathway in A549 and H1299 NSCLC cells [7]. Resveratrol also inhibits proliferation, migration, invasion and promotes apoptosis via inhibiting the messenger RNA (mRNA) and protein expression of signal transducer and activator of transcription 3 (STAT3) in A549 cells [8]. Resveratrol has been studied in phase I clinical trials, but most phase I studies have not been successful because of side effects and other unknown reasons [8–11]. Reports about new targets of resveratrol in NSCLC are still few and further investigations are urgently needed.

Cluster of differentiation (CD), also known as leukocyte differentiation antigen, which are often used as cellular markers for immune antigen recognition. CD molecules are used to distinguish between different lineages, different stages of differentiation and different levels of activation. In addition, mounting evidences have shown alterations in the expression level of CD molecules in many cancers. The above four CD antigens were all downregulated in NSCLC, but some other CDs have opposite effect in tumors. Wang et al. have found that mRNA and protein expression level of CD44v6 are significantly increased in malignant ovarian tumor tissues, compared with normal ovarian tissues. Meanwhile, experiments in ovarian cancer cell lines have proved that CD44v6 positively regulates cell migration, invasion and clone formation by activating nuclear factor kappa-B (NF-κB) pathway [12]. Another data from a meta-analysis reveals that high expression of CD44 indicates poorer survival and higher potential of metastasis in osteosarcoma patients [13]. It suggests one or several specific CDs may be as diagnostic markers for certain cancers and strategies of target CDs would be a viable way for cancer treatment.

The traditional single studies are limited by the finite number of samples and cannot systematically analyze the key genes and their molecular functions in complex biological processes. Whereas bioinformatics analysis based on high throughput platforms, is a powerful tool to identify meaningful genes as biomarker in cancer for diagnosis or prognosis. One previous study found that resveratrol inhibited NSCLC H1299 cell proliferation and induced cell senescence by leading to mitochondrial dysfunction and increasing of reactive oxygen species (ROS) [14]. Another study reported the effect of resveratrol on spontaneous tumors in individuals. Data in the annual fish *Nothobranchius guentheri* suggested that resveratrol inhibited age-dependent spontaneous tumorigenesis by increasing the expression of SIRT1 and activating its downstream targets [15]. Microarray (GSE9008) has been performed to evaluate the effect of resveratrol on A549 cells [16]. In this study, we performed a deeply bioinformatics analysis to predict the candidate genes effected by resveratrol treatment in NSCLC. By compared GSE9008 and GSE29250 datasets, 271 DEGs were selected. Functional enrichment analyses and protein-protein interaction (PPI) network of potential genes were performed and 10 hub genes were chosen. Finally, we identified *ANPEP*, *ITGAL*, *CD69*, and *PTPRC* as candidate genes associated with treatment of resveratrol in NSCLC via expression and survival analysis of hub genes.

## MATERIALS AND METHODS

### Data preparation

Expression data of mRNA for Non-small Cell Lung Cancer (NSCLC) patients was downloaded from NCBI-GEO (GSE29250). Dataset GSE29250 was based on GPL8179 and GPL10558 Platforms [Illumina Human v2 MicroRNA expression beadchip and Illumina HumanHT-12 V4.0 expression beadchip]. Expression data of human A549 mRNA was downloaded from NCBI-GEO (GSE9008), A549 cells were treated with 25 μM resveratrol for 48 hours and gene expression profiles were compiled using an oligonucleotide microarray to determine altered expression levels. It was based on GPL570 Platforms ([HG-U133_Plus_2] Affymetrix Human Genome U133 Plus 2.0 Array). The two datasets were analyzed with GRO2R. The *t*-test was applied to filter the DEGs, which were selected according to the original *P*-values <0.05 and fold change >1, and then DEGs in two datasets were merged and selected as potential targets for the further analysis.

### Data analysis

To assess functional enrichment, Gene ontology (GO) biological processes, cellular component and molecular function of DEGs were performed using Database for Annotation, Visualization, and Integration Discovery (DAVID) (https://david.ncifcrf.gov/home.jsp) [17]. The STRING (version 11.0) online database (https://string-db.org) was used to construct the protein-protein interaction (PPI) network [18]. Then the PPI network was visualized with Cytoscape software (version 3.6.0), and cytoHubba, a plugin of Cytoscape was used to calculate the key targets (hub genes) from the network [19]. There are 11 topological analysis methods in cytoHubba, and we chose Maximal Clique Centrality (MCC), which is based on the discovery of featured nodes and edges, and performs better than the others method in yeast PPI network [19]. To increase the sensitivity and specificity the intuition behind MCC is that essential proteins tend to be clustered in a yeast protein-protein interaction network [19]. Given a node *v*, the MCC of *v* is defined as $\text{MCC}(v)=\sum_{\text{C}\in \text{S(}v\text{)}}(|\text{C}|-1)!,$ where S(*v*) is the collection of maximal cliques which contain *v*, and (**|**C**|**−1)! is the product of all positive integers less than **|**C**|**. If there is no edge between the neighbors of the node *v*, then MCC(*v*) is equal to its degree. Each gene got a score according to the MCC mode, and we selected ten genes with the highest scores.

To confirm the expression of hub genes in clinical samples, we screened the expression of the 10 hub genes in the online platform, Gene Expression Profiling Interactive Analysis (GEPIA), which was a web server for analyzing the RNA sequencing expression data of 9,736 tumors and 8,587 normal samples from the TCGA and the GTEx projects, using a standard processing pipeline. A *P*-value <0.01 and a fold change >1 were considered as the threshold with expression between tumor and normal samples.

Furthermore, we did sample Meta analysis of hub genes with Oncomine™ (https://www.oncomine.org/resource/login.html). Six datasets of lung adenocarcinoma (LUAD) and four datasets of squamous cell lung carcinoma (LUSC) were selected to perform the Meta analysis. A *P*-value <1e-4 and a fold change >2 were considered as the threshold with gene ranking in the top 10%. Oncomine™ datasets are composed of samples represented as microarray data measuring either mRNA expression or DNA copy number on primary tumors, cell lines, or xenografts, usually from published research.

Moreover, survival curves of hub genes survival between high-expression and low-expression patients were validated in Kaplan Meier plotter (http://kmplot.com/analysis/). The Kaplan Meier plotter was a meta-analysis based tool to discover and validate of survival biomarkers. It was capable to assess the effect of 54k genes on survival in 21 cancer types. The largest datasets included breast (*n* = 6,234), and lung (*n* = 3,452) cancer [20].

Gene Set Cancer Analysis (GSCA) database was a web-based analysis platform for gene set cancer analysis (http://bioinfo.life.hust.edu.cn/web/GSCALite/). The platform integrated cancer genomics data of 33 cancer types from TCGA as well as normal tissue data from GTEx. The hub genes validated by Oncomine™ and Kaplan Meier plotter were further analyzed by GSCA for genetic alterations and methylation [21].

## RESULTS

### Data mining

**Figure 1:**
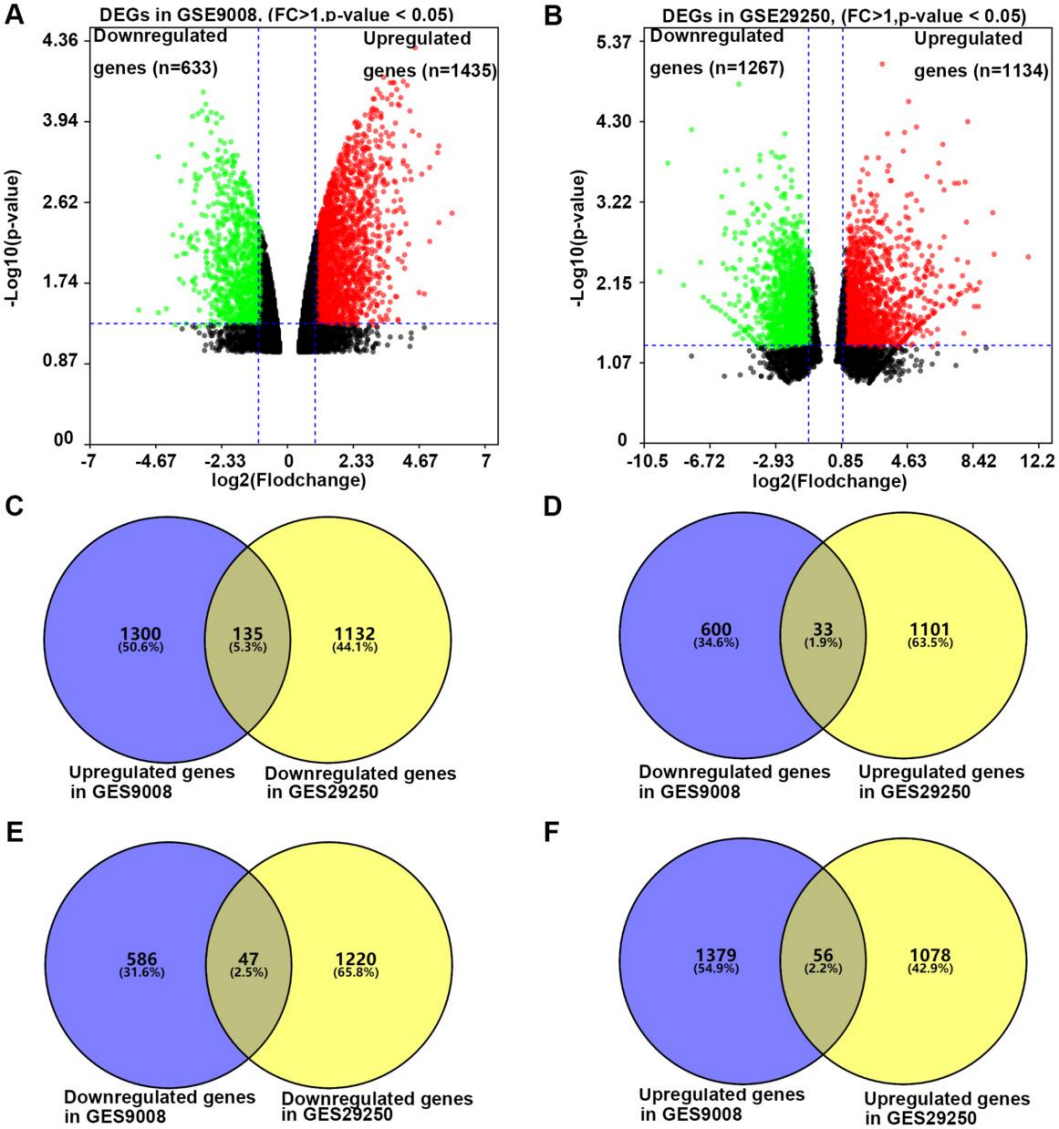
**The process of screening candidate genes.** (**A**) The DEGs in A549 cells treated with or without 25 microM resveratrol for 48 hours. Fold change >1 and *p*-value <0.05 was considered to be significant. (**B**) The DEGs in NSCLC tissue and adjacent normal tissue. Fold change >1 and *p*-value <0.05 was considered to be significant (**C**) Genes down-regulated in NSCLC and unregulated by resveratrol in A549 (*n* = 135). (**D**) Genes unregulated in NSCLC and down-regulated by resveratrol in A549 (*n* = 33). (**E**) Genes down-regulated in both NSCLC and resveratrol treated A549 (*n* = 47). (**F**) Genes upregulated in both NSCLC and resveratrol treated A549 (*n* = 56).

To investigate the effect of resveratrol on the expression of DEGs in NSCLC, we analyzed the dataset GSE29250 and dataset GSE9008 from NCBI-GEO. GSE29250 was an integrated analysis of miRNA and mRNA expressions of NSCLC by compared 12 NSCLC samples and their adjacent normal tissues. It contained 2401 DEGs (*P* < 0.05 and fold change >1), 1134 genes were up-regulated, while 1267 genes were down-regulated (Figure 1A). Data of GSE9008 were from one group of control NSCLC cell line-A549 and three groups of resveratrol treated A549. This dataset contained 2069 DEGs (*P* < 0.05 and fold change >1), 1436 genes were up-regulated, while 633 genes were down-regulated after (Figure 1B). Compared with the two groups of DEGs, 271 DEGs were selected for the further analysis. Of the 271 DEGs, 135 genes were down-regulated in NSCLC compared with adjacent tissue, and resveratrol treatment up-regulated these genes expression in A549 cells. Thirty-three genes were up-regulated in NSCLC compared with adjacent tissue, and resveratrol treatment reversed these genes expression (Figure 1C and 1D). All these 168 genes might be candidate genes of resveratrol which was beneficial for the treatment of NSCLC cancer. Meanwhile, 47 genes were found down-regulated in NSCLC, while decreased by resveratrol, too (Figure 1E). 56 genes were increased in NSCLC and the expressions were even higher after treatment with resveratrol (Figure 1F). These changes in gene expression may have the opposite effect or side effects in the treatment with resveratrol. Taken together, 271 DEGs were selected for the effect of resveratrol on NSCLC.

### Gene ontology analysis

To understand the function of the 271 DEGs, DAVID database was used to perform gene ontology analysis. As shown in Table 1, biological processes of DEGs were significantly enriched in the regulation of “signal transduction”, “positive regulation of transcription from RNA polymerase II promoter”, “cell adhesion”, “inflammatory response”, “negative regulation of cell proliferation”, and “cell surface receptor signaling pathway”. As to cellular component, the DEGs were significantly enriched in “cytoplasm”, “plasma membrane”, “extracellular exosome”, “integral component of plasma membrane”, “extracellular space”, and “cell surface”. About molecular function, the DEGs were significantly enriched in “calcium ion binding”, “protein homodimerization activity”, and “receptor activity”.

**Table 1:** Functional enrichment analysis of DEGs.

	**Term**	**Description**	**Count**	***P*-Value**
BP	GO:0007165	Signal transduction	37	4.04E-06
	GO:0045944	Positive regulation of transcription from RNA polymerase II promoter	23	0.0173101
	GO:0007155	Cell adhesion	18	2.25E-04
	GO:0006954	Inflammatory response	12	0.01637743
	GO:0008285	Negative regulation of cell proliferation	11	0.0489155
	GO:0007166	Cell surface receptor signaling pathway	10	0.01416262
CC	GO:0005737	Cytoplasm	83	0.03877089
	GO:0005886	Plasma membrane	81	9.98E-05
	GO:0070062	Extracellular exosome	58	5.01E-04
	GO:0005887	Integral component of plasma membrane	39	2.19E-05
	GO:0005615	Extracellular space	27	0.03352491
	GO:0009986	Cell surface	20	1.14E-04
MF	GO:0005509	Calcium ion binding	21	0.00217256
	GO:0042803	Protein homodimerization activity	17	0.04504788
	GO:0004872	Receptor activity	10	0.00315823

Abbreviations: BP: Biology Pathway; CC: Cellular Component; MF: Molecular Function.

### PPI network construction and identification of hub genes

**Figure 2:**
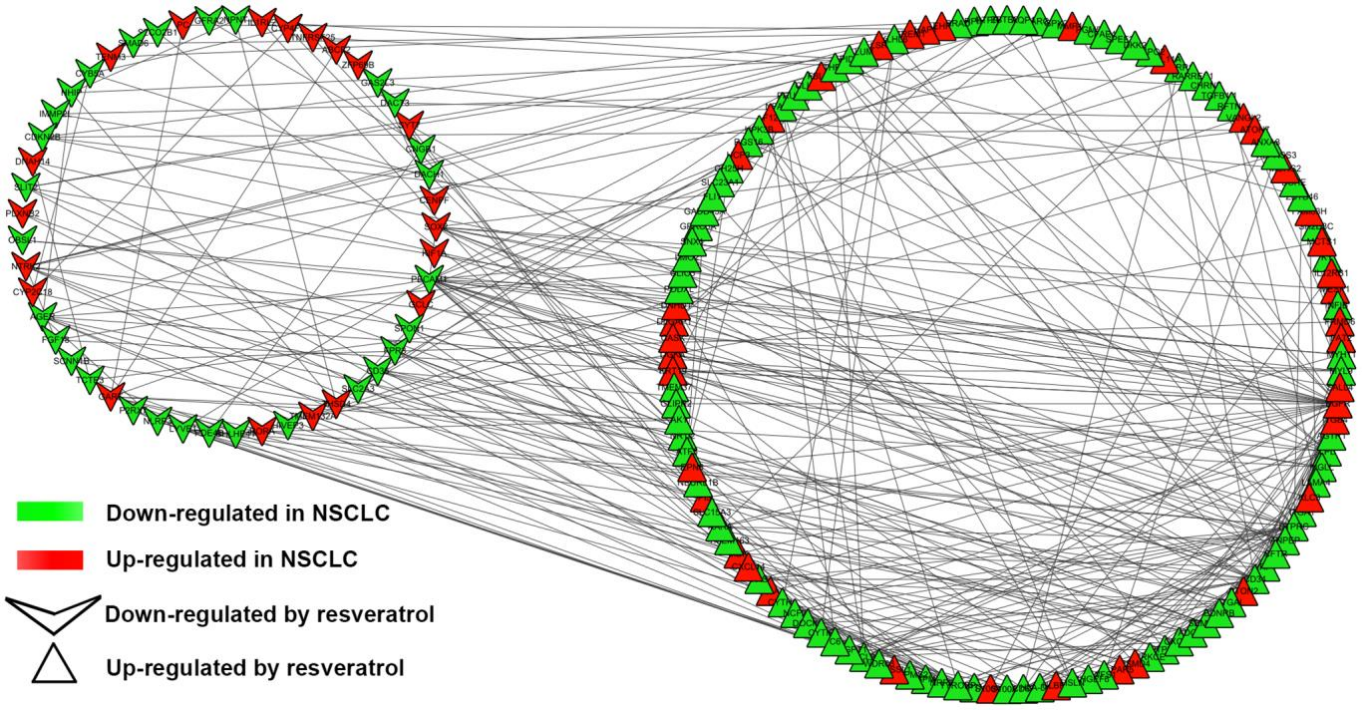
**PPI network of DEGs.** PPI network exported from STRING and visualized in Cytoscape. A total of 177 nodes and 342 edges were shown in the PPI network (disconnected nodes were removed). A node represents a gene. The genes reduced in NSCLC tissue were shown in green color. The genes increased in NSCLC tissue were shown in red color; at the same time, the genes which down-regulated in A549 by resveratrol were posted in triangles, and the genes which up-regulated by resveratrol were posted in shape “V”.

**Figure 3:**
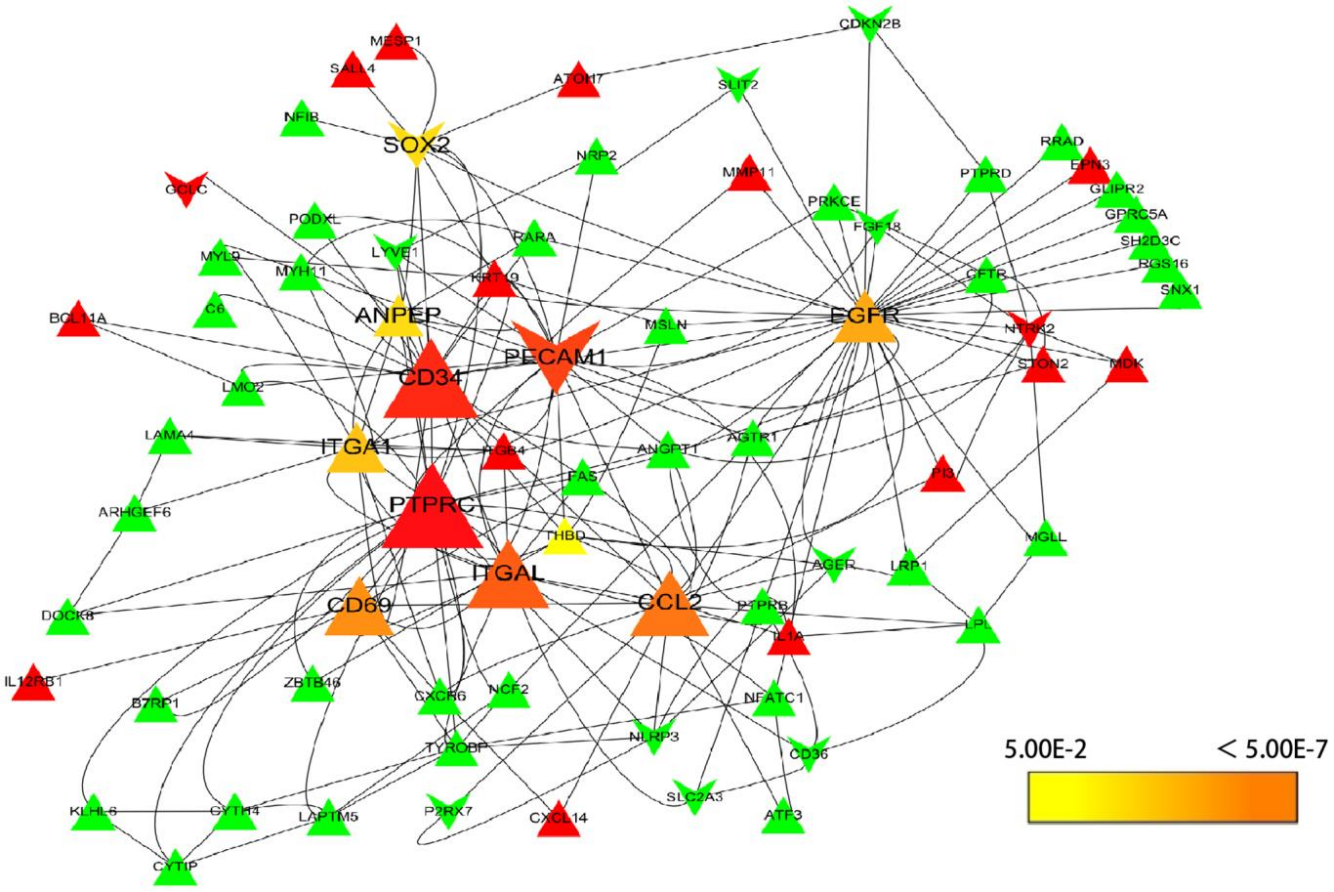
**Selection of hub genes.** PPI network was analyzed by cytohubba, a plugin of Cytoscape. A node represents a gene. The genes reduced in NSCLC tissue were shown in green color. The genes increased in NSCLC tissue were shown in red color; at the same time, the genes which down-regulated in A549 by resveratrol were posted in triangles, and the genes which up-regulated by resveratrol were posted in shape “V”. The hub genes with higher scores were demonstrated with larger size.

To understand the interaction of DEGs at protein level, the 271 DEGs were analyzed in STRING, 255 nodes and 342 edges were shown in the PPI network (PPI enrichment *P*-value <1.0e-16). Cytoscape was used to visualize and analyze the PPI network. After removing the disconnected nodes, 177 nodes and 342 edges were shown in Figure 2. CytoHubba, a plugin of Cytoscape was used to calculate the hub genes from the network. *ANPEP*, *CD69*, *ITGAL*, *PECAM1*, *PTPRC*, *CD34*, *ITGA1*, *CCL2*, *SOX2*, and *EGFR* were identified as the hub genes of the network according to the high scores (Figure 3). Hub gene ranks were shown in Supplementary Table 1. In these genes, the mRNA levels of *ANPEP*, *CD69*, *ITGAL*, *PTPRC*, *CD34*, *ITGA1*, and *CCL2* were reduced in NSCLC and up-regulated by resveratrol treatment. *PECAM1* was also down-regulated in NSCLC and the expression was even lower by resveratrol treatment. Both *EGFR* and *SOX2* were up-regulated in NSCLC; resveratrol treatment promoted *EGFR* and inhibited *SOX2*. In this condition, we predicted that the regulation of resveratrol on *ANPEP*, *CD69*, *ITGAL*, *PTPRC*, *CD34*, *ITGA1*, *CCL2*, and *SOX2* expression were beneficial to the treatment of NSCLC. However, it was worth paying attention to the effect of resveratrol treatment on *PECAM1* and *EGFR*.

### Validation hub genes in clinical samples

**Figure 4:**
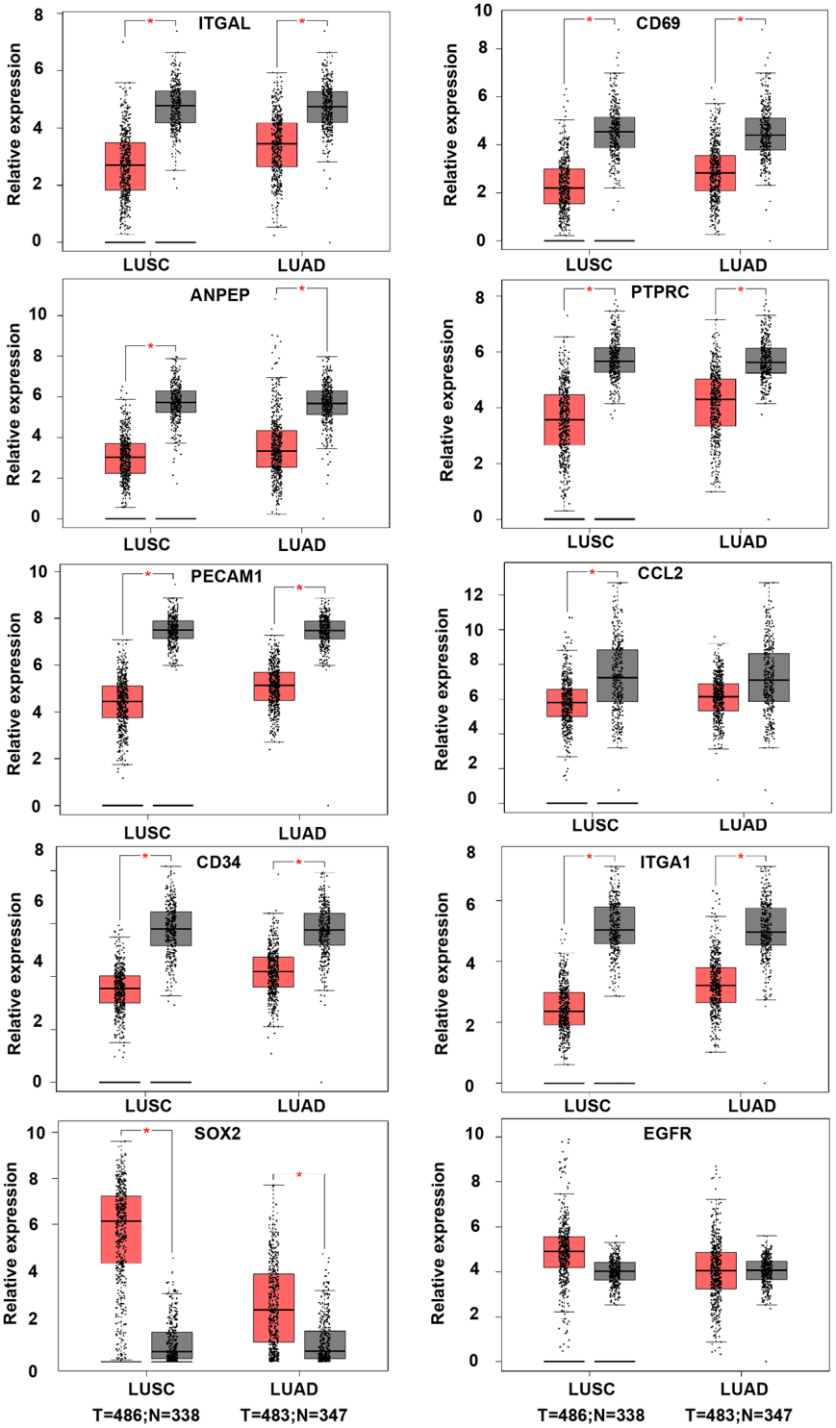
**The expression of hub genes in LUSC and LUAD samples of TCGA.** The LUAD database was containing 486 tumor and 338 normal samples. LUAD datasets was containing 483 tumor and 347 normal samples. *ANPEP, CD69, ITGAL, PTPRC, ITGA1, CCL2*, and *PECAM1* were down-regulated in both LUSC and LUAD samples. SOX2 was up-regulated in both LUSC and LUAD samples. There was no significant different on EGFR expression between tumor and normal samples (^*^P < 0.05).

**Figure 5:**
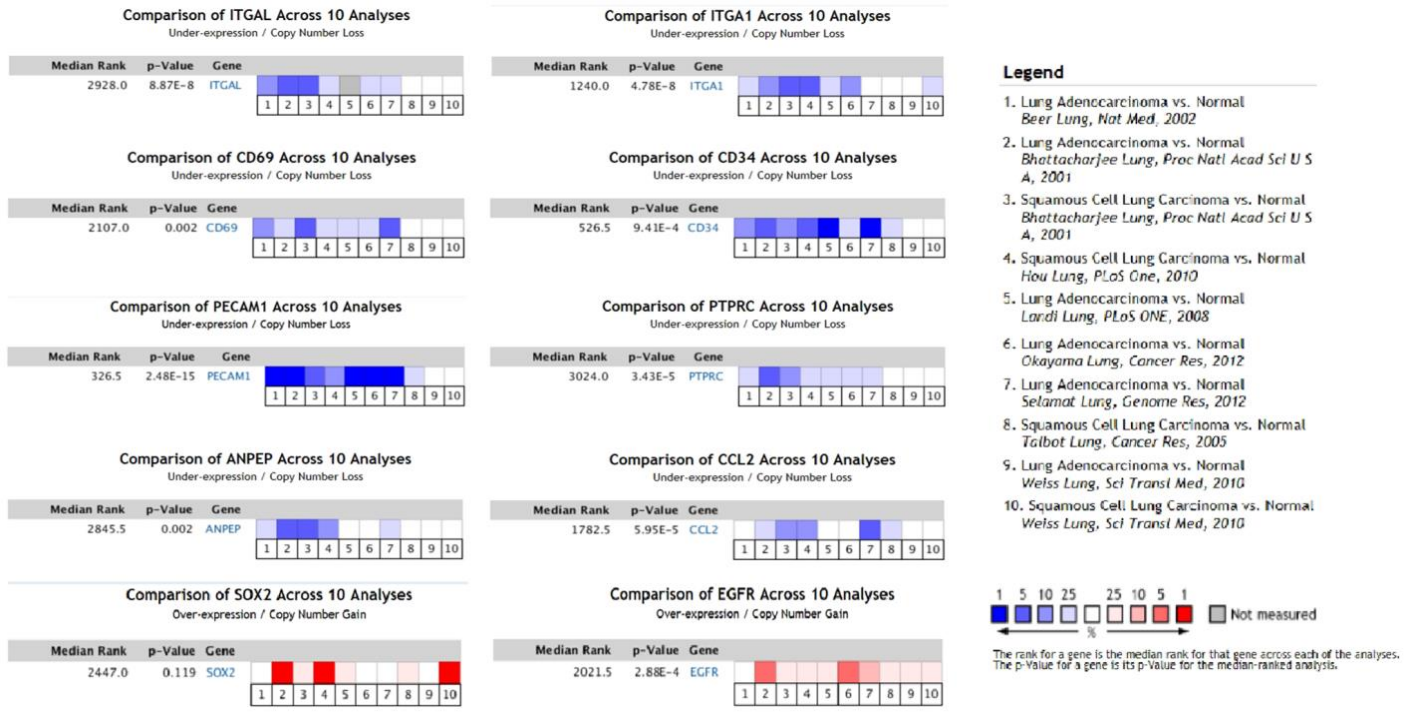
**The expression of hub genes in Oncomine™.** Six LUAD and four LUSC datasets were selected and the expression of the hub genes was compared across the ten datasets. Normal tissues were chosen as control. Blue colors represented under-expression, and red colors referred to over-expression of hub genes. Colors were shown in blue or red from light to dark according to the quantity of expression (from low to high). *ANPEP, CD69, ITGAL, PTPRC, ITGA1, CCL2*, and *PECAM1* were considered to down-regulate in LUSC and LUAD datasets (*P* < 0.05). EGFR was up-regulated in LUSC and LUAD datasets (*P* < 0.05). There was no significant different on SOX2 expression between tumor and normal samples (*P* = 0.119).

**Figure 6:**
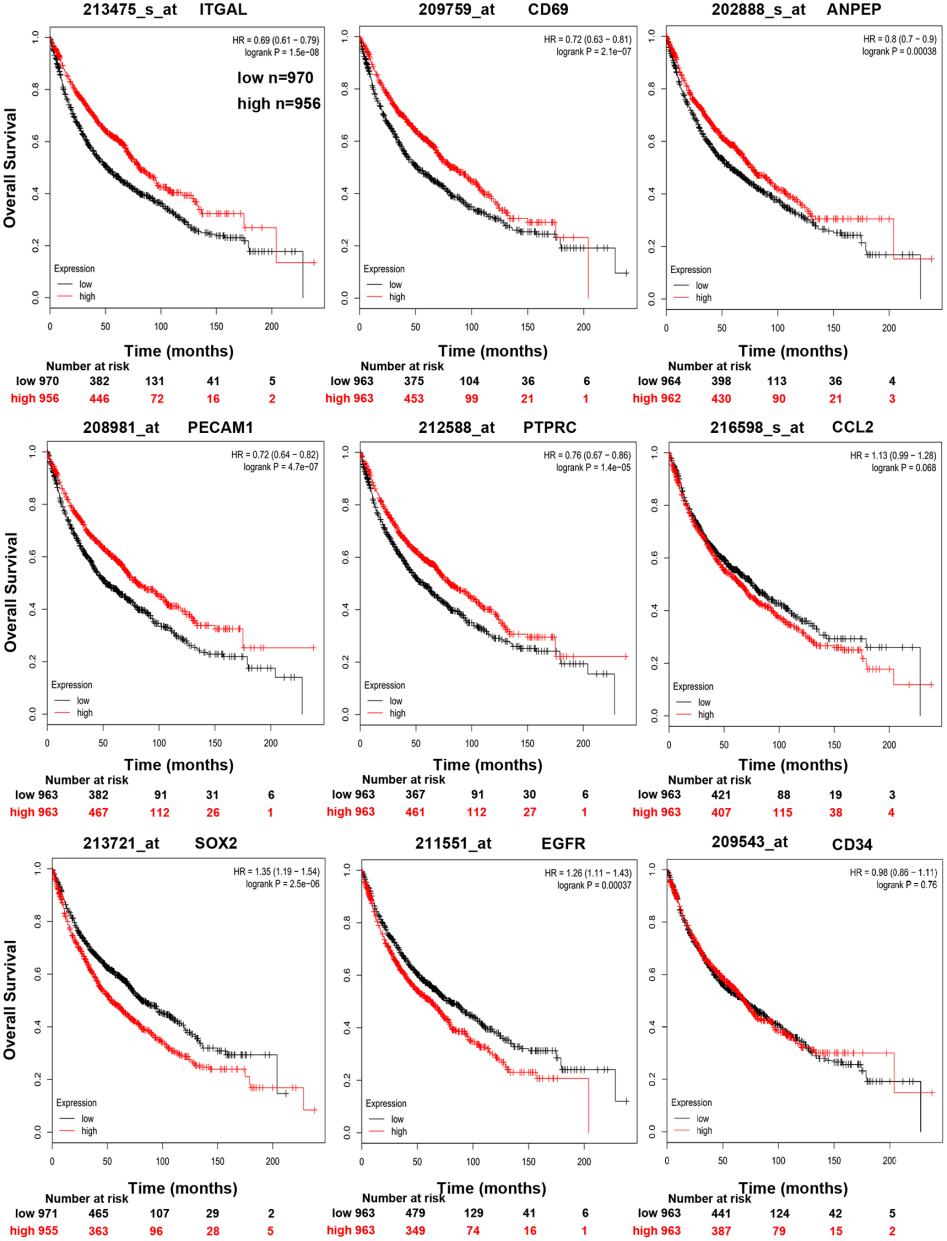
Kaplan-Meier curves displaying OS of NSCLC with high and low expression of *ANPEP, CD69, ITGAL, PTPRC, CCL2, PECAM1, CD34, SOX2*, and *EGFR*.

In order to validate whether the DEGs identified were also abnormally expressed in NSCLC, we determined the expression of hub genes in clinical samples of LUAD and LUSC from GEPIA and Oncomine™. With the exception of *EGFR*, the expression trends of the other genes were as same as our analysis in LUAD and LUSC samples compared with normal samples (*P* < 0.05) (Figure 4). Moreover, the expression of DEGs was analyzed in ten datasets (Figure 5). The *P* value of *SOX2*, *EGFR*, and CD34 were higher than 0.05, the expression trends of *ANPEP, CD69, ITGAL, PTPRC, ITGA1, CCL2*, and *PECAM1* were as same as our analysis in LUAD and LUSC samples compared with normal samples (*P* < 0.05). To determine the effect of DEGs on prognosis of lung cancer patients, Kaplan-Meier plotter was used to predict the prognostic value of 10 hub genes. Our results found that low expression of *PECAM1* was associated with worse overall survival (OS) of NSCLC patients, as well as *ANPEP, CD69, ITGAL*, and *PTPRC* (*P* < 0.05) (Figure 6). Additionally, high expression of *SOX2* and *EGFR* was associated with worse OS of NSCLC patients (*P* < 0.05) (Figure 6). The expression of *CCL2* and *CD34* had no associated with OS of NSCLC patients (*CCL2, P = 0.068; CD34, P = 0.76*). Unfortunately, the result of *ITGA1* on OS of NSCLC patients was not retrieved in the Kaplan-Meier plotter database. It was interesting that high expression of *PECAM1* was associated with better OS. However, in our study *PECAM1* was decreased in NSCLC patients, and resveratrol treatment reduced the expression of *PECAM1.*

### Single nucleotide variation, methylation and pathway activity of validated genes

**Figure 7:**
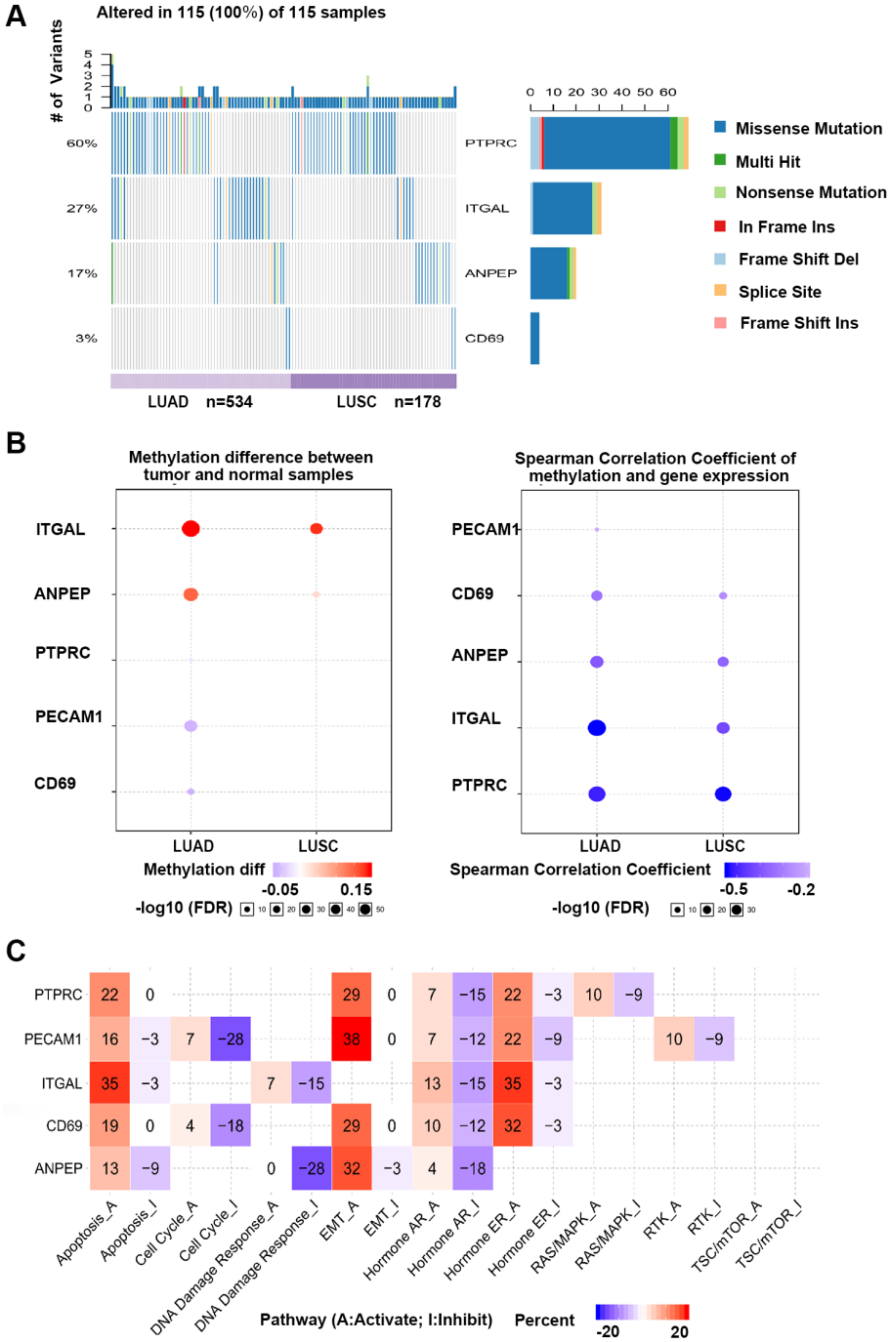
**GSCA online database was selected to analyze the single nucleotide variation, methylation and pathway activity of validated genes.** (**A**) Waterfall plots, gives a single nucleotide variation of in LUAD and LUSC gene sets. (**B**) Methylation of hub genes, Blue points represented a methylation upregulation in tumors, red points represented a methylation downregulation in tumors, the deeper of color, the higher the difference. Student *T* test were performed to define the methylation difference between tumor and normal samples. The association between mRNA expression and methylation was based on Person’s product moment correlation coefficient, and followed a t distribution. Blue points represented negative correlation, and red represented positive correlation, the deeper of color, the higher the correlation. *P* value was adjusted by false discovery rate (FDR), FDR ≤ 0.05 was considered as significant. Size of the point represents statistic significance (**C**) Effects of validated gene on cell pathway activity, gene expression was divided into 2 groups (High and Low) by median expression, the difference of pathway activity score (PAS) between groups was defined by student *T* test, *P* value was adjusted by FDR, FDR ≤ 0.05 was considered as significant. Pathway Activity module presents the correlation of genes expression with pathway activity groups (activation and inhibition) that defined by pathway scores.

According to the results verified in clinical samples, *ANPEP, CD69, ITGAL*, and *PTPRC* were considered as candidate genes and analyzed in GSCA database. Single nucleotide variations (SNV) of 4 hub genes were detected in 115 of 712 samples (Figure 7A). The SNV frequency of *PTPRC* was the highest among 4 hub genes, reaching 60% in 115 samples. Missense mutation was the most important mutation type. Gene expression was significantly influenced by genome methylation, we also detected the methylation difference of hub genes in cancer and normal samples. As shown in Figure 7B, methylation of *ITGAL* and *ANPEP* were down-regulated, while methylation of *CD69* and *PTPRC* were up-regulated in cancer. We further evaluated the effect of methylation on hub gene expression, and found that methylation of all 4 hub genes was negatively correlated with gene expression. This result demonstrated that down-regulation of *CD69* and *PTPRC* in NSCLC might be regulated by methylation, and other mechanism should be considered to regulate expression of *ITGAL* and *ANPEP* in NSCLC. Effect of hub genes on pathway activity has also been considered in the work (Figure 7C). Hub genes were involved in the regulation of apoptosis, cell cycle, EMT, and hormone signal pathway, which are important signal pathways for tumor genesis and development.

## DISCUSSION

Resveratrol is a phytoestrogen that has been reported to possess wide biological effects, such as anti-oxidant, anti-inflammatory, cardioprotective, and anti-cancer properties [22]. Resveratrol suppresses cancer growth, induces cancer cell apoptosis, and inhibits tumor angiogenesis and metastasis [23]. Although some signal pathways mentioned above have explained the anti-cancer effects of resveratrol, the full mechanisms of resveratrol interfere with cancer are not defined in NSCLC [24].

In this study, a total of 271 DEGs were identified, including 135 genes were increased by resveratrol treatment and down-regulated in NSCLC, 33 genes were reduced by resveratrol and up-regulated in NSCLC. 47 genes were found decreased by resveratrol and down-regulated in NSCLC, 56 genes were increased in NSCLC and the expressions were even higher after resveratrol treatment. In these genes, *ANPEP, CD69, ITGAL, PECAM1, PTPRC, CD34, ITGA1, CCL2, SOX2*, and *EGFR* were identified as key DEGs in the treatment of resveratrol in NSCLC, then expression analysis and survival analysis of these ten genes demonstrated that *ANPEP, CD69, ITGAL*, and *PTPRC* were the potential candidate genes significantly correlated with resveratrol treatment in NSCLC. Resveratrol treatment increased expression of *ANPEP, CD69, ITGAL* and *PTPRC*. The relationship between potential candidate genes expression and methylation was analyzed. As showed in Figure 7, methylation of *ANPEP*, and *ITGAL* in tumor cells was elevated compared with normal tissue. DNA methylation of ANPEP, CD69, ITGAL, and PTPRC are negative related to the gene expression. The expression of PTPRC was negatively correlated with the promoter methylation levels in all hematological cells [25]. Knockout of the lysine methyltransferase KMT1E (Setdb1) in thymocyte caused the reduction of CD69 in T cells [26]. It has been reported that the methylation patterns of ITGAL contributed to the development of autoimmunity, aging and cancer [27]. In Jurkat cells, spermine treatment inhibited lymphocyte function by increasing DNA methyltransferase, which enhanced demethylation of the ITGAL promoter and reduced ITGAL expression [28]. The expression of ANPEP was high associated with DNA methylation status of the ANPEP promoter in melanoma cells [29]. Inhibition of DNA methyltransferase increased ANPEP expression and reduced APN-dependent migration of melanoma cells [29]. In prostate cancer patients, low expression and aberrant promoter hypermethylation of ANPEP have been considered as a new independent adverse prognostic factor for patients [30]. Previous studies revealed that resveratrol treatment induced epigenetic changes in many diseases including cancer [31]. Resveratrol treatment has inhibited DNA methyltransferases (DNMTs) including DNMT1, DNMT3a, and DNMT3b expression and activity in human cancer cells [32, 33]. In NSCLC, resveratrol inhibited the expression of DNA (cytosine-5)-methyltransferase 1 and induced demethylation of the DNA (cytosine-5)-methyltransferase 1 target gene [34]. All these evidences suggest that resveratrol affects gene expression by regulating of methylation.

Both protein tyrosine phosphatase receptor type C (PTPRC, also known as CD45) and integrin alpha L chain (ITGAL, also known as CD11a) are pan-leukocyte markers. PTPRC participates in various biological processes including cell growth, mitosis, and malignant transformation [35]. The expression of *PTPRC* is highly associated with the methylation in testicular germ cell tumors (TGCT) [36]. It has been reported that PTPRC was associated with LUAD patients overall survival and recurrence free survival time [37]. Data of liver biopsy from 65 primary tumor patients showed that positive PTPRC staining in liver tissue was associated with better cumulative survival rate [38]. This study is consistent with our results herein NSCLC, that high expression of *PTPRC* was associated with better overall survival for NSCLC patients. In dataset GSE9008, resveratrol treatment increased mRNA level of PTPRC in A549 cells, which could be the potential target of resveratrol inhibit tumor progress. PTPRC negatively regulates STAT3 activities by dephosphorylation of JAKs [39]. STAT3 is crucial for tumor development and as a target for anti-tumor therapy [40]. Meanwhile resveratrol has been proved to inhibit STAT3 activity in lung cancer [41]. However, the mechanisms of resveratrol inhibit STAT3 activity via regulating PTPRC need to be further explored. ITGAL forms LFA-1 with ITGB2 [42]. LFA-1 participated in cell-interaction, adhesion and cytotoxic T-cell killing [43]. The anti-tumor role of ITGAL has been confirmed in fibrosarcoma [44]. ITGAL combined with other five peripheral blood derived genes has been used to predicate OS in cancer patients [43]. Another study has been reported that SNPs of ITGAL was associated with high risk of death in castration-resistant prostate cancer (CRPC); there was no substantial relation between SNPs and ITGAL expression [45]. In adenocarcinoma patients, ITGAL was associated with N stage of tumor [46].The effects of resveratrol on ITGAL were less reported. Previous study showed that chemotherapy with propranolol and etodolac increased ITGAL expression and improved recurrence-free survival rates in mice undergoing primary melanoma or Lewis lung carcinoma excision [47]. This result suggested that high level of ITGAL was beneficial for the survival of mice models and was also consistent with our result that increased expression of ITGAL was associated with better overall survival (OS) for NSCLC patients.

CD69 is a type-2 glycoprotein and involved in inflammatory diseases including cancer by interacting with nonspecific legends [48]. The role of CD69 in cancer therapy is ambiguous. It has been reported that lost of CD69 enhanced anti-tumor immunity in CD69^−/−^ mice, CD69^−/−^ mice or CD69 antibody significantly inhibited RM-1 prostate carcinoma growth and metastasis [49, 50]. The expression of CD69 caused retention of tumor-infiltrating CD8 T cells and increased TGFβ production, and these may be the main incentives that induced exhaustion of CD8 T cells [49, 50]. However, during tumor progression, lymphocytes were damaged in tumor microenvironment, and tumor infiltrating lymphocytes (TILs) with CD103^+^CD8^+^CD69^+^ were important for eliminating of tumor cells. Increased TILs with CD69^+^ were a positive prognostic marker in NSCLC models [51]. Clinical research demonstrated that the level of CD44^+^, CD54^+^, and CD69^+^ lymphocytes in peripheral blood of lung cancer patients were significantly lower than the health and rose after chemotherapy [52]. Treatment with plasma of lung cancer patients inhibited lymphocytes proliferation and reduced CD69 expression, which caused suppression of lymphocyte activity [53]. These results consisted with our finding that CD69 was lower expressed than adjacent tissue. Resveratrol has been reported to increase CD69 expression by inhibiting expression of BCL-6 and subsequently induced apoptosis of transformed follicular lymphoma [54]. It is worth noting that resveratrol also inhibited TGF-β1 expression and substantially prevented TGF-β1/SMADs signal pathway [55], which suggested that resveratrol may prevent exhaustion of CD8^+^ T cells by inhibiting TGF-β1 signal pathway. Indeed, it has been reported that resveratrol combined treatment with anti-tumor drugs sensitized leukemic lymphocytes and protected normal lymphocytes [56, 57].

Aminopeptidase N (ANPEP or CD13) was a specificity aminopeptidase which had an important role in angiogenesis [58] and participated in drug resistance via regulating ephrine receptor A2 in melanoma cells [59]. The target role of ANPEP on cancer therapy has been summarized before, and evaluation of ANPEP could be a diagnostic and prognostic factor in solid malignancies [60]. We found that mRNA level of *ANPEP* was down-regulated and the promoter of ANPEP was hypermethylated in NSCLC samples. Down-regulation of *ANPEP* was associated with worse overall survival for NSCLC patient in Kaplan Meier plotter (*n* = 1926). Consistent with previous reports that low expression and hypermethylation of ANPEP were correlated inversely with survival in prostate cancer [30]. Of interesting, the soluble form of ANPEP was secreted into serum and the plasma levels of ANPEP were increased in patients with NSCLC [61]. High serum ANPEP was associated with worse tumor progression stage and overall survival rate [61]. The studies using immunohistochemical assay showed that the expression of ANPEP was positive in one-third of the patients and 5-year survival rate was lower than ANPEP negative patients [62]. Another study containing 96 patients demonstrated that the expression of ANPEP was positive only in 9% cases, and there was no significant difference of 5-year survival rate between groups based on ANPEP status [63]. Recently, studies about 270 NSCLC patients reported that ANPEP expression in endothelial cells, vessel-associated stroma cells and tumor cells had no prognostic effect in entire NSCLC [64]. However, ANPEP expression in endothelial cell and vessel-associated stroma cell had positive prognostic effect on NSCLC patients with squamous cell carcinoma, and with negative prognostic effect on tumor stage III patients, and for patients with pN2 lymph node status. In this study univariate prognostic models showed that tumor cells ANPEP mRNA expression was associated with increased over-all survival. They attributed discrepancies, about the effect of ANPEP on prognostic effect and survival, between different groups to heterogeneity in patient cohorts or methodology [64]. Though results which compared the mRNA data with protein data were contradictory, post-transcriptional regulation of ANPEP could explain. Resveratrol up-regulated mRNA level of ANPEP, which was positive associated with survival of NSCLC patients. However, the effect of resveratrol on the post-transcriptional regulation of ANPEP needs to be in-depth study.

In conclusion, we demonstrated that resveratrol mainly effects the molecular involved in signal transduction, cell adhesion, and inflammatory response. We found that *ANPEP*, *CD69*, *ITGAL*, and *PTPRC* are down-regulated in patients of NSCLC and the expression of these genes is regulated by DNA methylation. Resveratrol increases mRNA expression of the above four molecules. Data of clinical samples confirmed that high expression of these molecules were associated with better prognosis. We suggested that *ANPEP*, *CD69*, *ITGAL*, and *PTPRC* might be important targets associated with NSCLC treatment by resveratrol and the mechanism required further study.

## Supplementary Materials

Supplementary Table 1
