# Supplementary material for: Identification of potential target genes of non-small cell lung cancer in response to resveratrol treatment by bioinformatics analysis
Source: Aging (Albany NY). 2021 Oct 11;13(19):23245–61. doi: 10.18632/aging.203616 (PMC8544309; doi:10.18632/aging.203616)
Supplement: Supplementary Table 1 [file aging-13-203616-s001.pdf]

SUPPLEMENTARY TABLE

Supplementary Table 1. Top 10 genes in network string interactions ranked by MCC method [19].

Rank	Name	Score
1	PTPRC	880
2	CD34	845
3	PECAM1	702
4	ITGAL	496
5	CCL2	440
6	CD69	381
7	EGFR	344
8	ITGA1	286
9	SOX2	148
10	ANPEP	148
